# Error and bias in under-5 mortality estimates derived from birth histories with small sample sizes

**DOI:** 10.1186/1478-7954-11-13

**Published:** 2013-07-26

**Authors:** Laura Dwyer-Lindgren, Emmanuela Gakidou, Abraham Flaxman, Haidong Wang

**Affiliations:** 1 Institute for Health Metrics and Evaluation, University of Washington, Seattle, WA, USA

## Abstract

**Background:**

Estimates of under-5 mortality at the national level for countries without high-quality vital registration systems are routinely derived from birth history data in censuses and surveys. Subnational or stratified analyses of under-5 mortality could also be valuable, but the usefulness of under-5 mortality estimates derived from birth histories from relatively small samples of women is not known. We aim to assess the magnitude and direction of error that can be expected for estimates derived from birth histories with small samples of women using various analysis methods.

**Methods:**

We perform a data-based simulation study using Demographic and Health Surveys. Surveys are treated as populations with known under-5 mortality, and samples of women are drawn from each population to mimic surveys with small sample sizes. A variety of methods for analyzing complete birth histories and one method for analyzing summary birth histories are used on these samples, and the results are compared to corresponding true under-5 mortality. We quantify the expected magnitude and direction of error by calculating the mean error, mean relative error, mean absolute error, and mean absolute relative error.

**Results:**

All methods are prone to high levels of error at the smallest sample size with no method performing better than 73% error on average when the sample contains 10 women. There is a high degree of variation in performance between the methods at each sample size, with methods that contain considerable pooling of information generally performing better overall. Additional stratified analyses suggest that performance varies for most methods according to the true level of mortality and the time prior to survey. This is particularly true of the summary birth history method as well as complete birth history methods that contain considerable pooling of information across time.

**Conclusions:**

Performance of all birth history analysis methods is extremely poor when used on very small samples of women, both in terms of magnitude of expected error and bias in the estimates. Even with larger samples there is no clear best method to choose for analyzing birth history data. The methods that perform best overall are the same methods where performance is noticeably different at different levels of mortality and lengths of time prior to survey. At the same time, methods that perform more uniformly across levels of mortality and lengths of time prior to survey also tend to be among the worst performing overall.

## Background

Under-5 mortality, the probability of death before age 5 (denoted _5_*q*_0_), is an important overall indicator of child health. In countries without functioning systems to continuously register births and deaths, estimates of under-5 mortality are generally derived from survey and/or census data, particularly in the form of birth histories where women are asked for information about the survival of their children.

Birth history data are routinely used for estimating mortality at the national level. It is often of interest, however, to estimate under-5 mortality at a subnational level or to stratify by some other characteristic (e.g., income or maternal education). Subnational or stratified analyses with survey data are complicated by small sample sizes: in the case of surveys in particular, the sample size for a given subnational unit or stratum is often quite small, and it is not apparent if the estimates derived from these limited data are useful. While a number of subnational analyses with birth history data have been undertaken using census data [1-3] where small sample sizes are less of a concern, existing subnational mortality estimates using survey data tend to be at a relatively coarse level (often provinces or regions) to avoid small samples [2,4].

Two different types of birth histories are routinely collected. In a complete birth history (CBH), women are asked for information about the date of birth and, if applicable, the age at death of each child they have given birth to. Because complete birth histories contain information about dates and ages for individual children they allow for direct calculation of under-5 mortality. In a summary birth history (SBH), women are asked only about the total number of children they have given birth to and the number of these children who are still alive. Summary birth histories lack information about dates and ages for individual children and demographic models must be employed to estimate under-5 mortality from these data. Although complete birth histories are more straightforward to analyze they are less frequently undertaken than summary birth histories, which are far less labor-intensive and time-consuming to collect.

In this paper, we aimed to determine how much error and/or bias can be expected in under-5 mortality estimates derived from both types of birth histories at various small sample sizes. To this end, we carried out a data-based simulation study using Demographic and Health Survey (DHS) data wherein we treated each survey as a population with known mortality and sampled from this population to mimic surveys with small sample sizes. We examined how estimates derived from summary birth history data and complete birth history data (analyzed using several alternative methods) compared in terms of error and bias at increasingly small sample sizes. Further, we performed stratified analyses to explore in more detail how the performance of each method relates to the underlying true level of mortality and the time prior to data collection.

## Methods

### Data

This analysis made use of all DHS [5] publicly available as of May 2012 that contain birth histories for all women, regardless of marital status, a total of 152 surveys in 62 countries. Table 1 provides a full listing of all DHS included in this analysis.

**Table 1:** Demographic and health surveys included in this analysis

**Country**	**Survey years (sample size)**
Albania	2008-09 (7584)
Armenia	2000 (6430); 2005 (6566); 2010 (5922)
Azerbaijan	2006 (8444)
Benin	1996 (5491); 2001 (6219); 2006 (17794)
Bolivia	1989 (7923); 1993–94 (8603); 1998 (11187); 2003–04 (17654); 2008 (16939)
Botswana	1988 (4368)
Brazil	1986 (5892); 1996 (12612)
Burkina Faso	1992–93 (6354); 1998–99 (6445); 2003 (12476)
Burundi	1987 (3970)
Cambodia	2000 (15351); 2005–06 (16823); 2010–11 (18754)
Cameroon	1991 (3871); 1998 (5501); 2004 (10656)
Central African Republic	1994–95 (5884)
Chad	1996–97 (7454); 2004 (6085)
Colombia	1986 (5329); 1990 (8644); 1995 (11140); 2000 (11585); 2004–05 (41344)
Comoros	1996 (3050)
Congo	2005 (7051)
Congo, Democratic Republic of	2007 (9995)
Côte d’Ivoire	1994 (8099); 1998–99 (3040)
Dominican Republic	1986 (7645); 1991 (7318); 1996 (8422); 2002 (23384); 2007 (27195)
Ecuador	1987 (4713)
Eritrea	1995–96 (5054); 2002 (8754)
Ethiopia	2000 (15367); 2005 (14070); 2010–11 (16515)
Gabon	2000–01 (6183)
Ghana	1988 (4488); 1993–94 (4562); 1998–99 (4843); 2003 (5691); 2008 (4916)
Guatemala	1987 (5160); 1995 (12403); 1998–99 (6021)
Guinea	1999 (6753); 2005 (7954)
Guyana	2009 (4996)
Haiti	1994–95 (5356); 2000 (10159); 2005–06 (10757)
Honduras	2005–06 (19948)
Kazakhstan	1995 (3771); 1999 (4800)
Kenya	1988–89 (7150); 1993 (7540); 1998 (7881); 2003 (8195); 2008–09 (8444)
Kyrgyzstan	1997 (3848)
Lesotho	2004–05 (7095); 2009–10 (7624)
Liberia	1986 (5239); 2006–07 (7092)
Madagascar	1992 (6260); 1997 (7060); 2003–04 (7949); 2008–09 (17375)
Malawi	1992 (4849); 2000 (13220); 2004–05 (11698); 2010 (23020)
Mali	1987 (3200); 1995–96 (9704); 2001 (12849); 2006 (14583)
Mauritania	2000–01 (7728)
Moldova	2005 (7440)
Mozambique	1997 (8779); 2003 (12418)
Namibia	1992 (5421); 2000 (6755); 2006–07 (9804)
Nicaragua	1997–98 (13634); 2001 (13060)
Niger	1992 (6503); 1998 (7577); 2006 (9223)
Nigeria	1990 (8781); 2003 (7620); 2008 (33385)
Paraguay	1990 (5827)
Peru	1986 (4999); 1991–92 (15882); 1996 (28951); 2000 (27843); 2004–08 (41648)
Philippines	1998 (13983); 2003 (13633); 2008 (13594)
Rwanda	1992 (6551); 2000 (10421); 2005 (11321); 2007–08 (7313); 2010–11 (13671)
Sao Tome and Principe	2008–09 (2615)
Senegal	1986 (4415); 1992–93 (6310); 1997 (8593); 2005 (14602); 2010–11 (15688)
Sierra Leone	2008 (7374)
South Africa	1998 (11735)
Swaziland	2006–07 (4987)
Tanzania, United Republic of	1991–92 (9238); 1996 (8120); 1999 (4029); 2004–05 (10329); 2009–10 (10139)
Timor-Leste	2009–10 (13137)
Togo	1988 (3360); 1998 (8569)
Trinidad and Tobago	1987 (3806)
Uganda	1988–89 (4730); 1995 (7070); 2000–01 (7246); 2006 (8531)
Ukraine	2007 (6841)
Uzbekistan	1996 (4415)
Zambia	1992 (7060); 1996–97 (8021); 2001–02 (7658); 2007 (7146)
Zimbabwe	1988–89 (4201); 1994 (6128); 1999 (5907); 2005–06 (8907); 2010–11 (9171)

### Birth history methods

#### Summary birth history method

We analyzed summary birth history data using updated models and methods described in Rajaratnam, et al. [6,7]. The combined version of the maternal age cohort, time since first birth cohort, maternal age period, and time since first birth period methods was used to generate annual estimates for the 25 years preceding each survey.

#### Standard complete birth history method

To analyze complete birth history data we first expanded the record for each child such that there was a record of each month that a child lived and was observed under age 5: this will be less than the full 60 months if the child died before age 5 or if the mother was surveyed before the child reached age 5. For each child-month of life we indicated whether the child was alive or dead at the end of the month and then assigned the child-month to the appropriate time period and age group. Time periods were non-overlapping and equally sized and were assigned starting at the time of the most recent survey and moving back in time. The ages considered were 0 months, 1–11 months, 12–23 months, 24–35 months, 36–47 months, and 48–59 months; these age groupings were designed such that mortality is expected to be reasonably constant across the age range. From these data we calculated the monthly probability of survival in each time period for each age group by calculating the proportion of child-months in a given time period and age group that end with the child alive. These monthly probabilities of survival were converted to the probability of surviving the entire age interval under consideration by raising them to a power equal to the number of months in the age interval. Under-5 mortality was then calculated by subtracting from one the product of all of the age-specific survival probabilities. This process generated a single estimate of under-5 mortality for each time period which was then assigned to the midpoint of the period. Different length periods can be used, with longer periods providing more pooling of information across time but also producing less frequent estimates. For this analysis, we tested periods of length one, two, and five years. It is possible to pool data from multiple surveys in the same country and estimate mortality from the combined data [8]. Except when explicitly stated otherwise, the non-pooled version of the complete birth history method is used throughout this analysis.

#### Moving window complete birth history method

**Figure 1:**
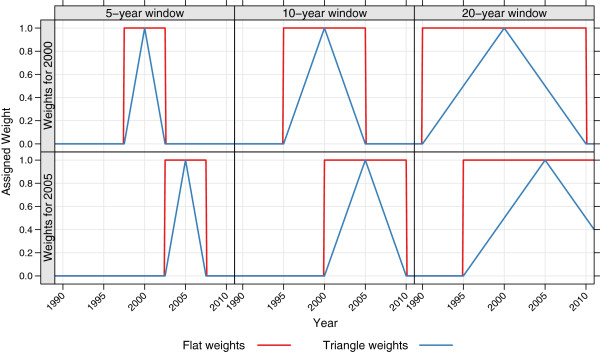
**Demonstration of weighting scheme used for moving window variants of complete birth history methods.** Each panel shows the weights assigned to child-months in each month between 1990–2010 when predicting for a given year (2000 in the top row and 2005 in the bottom row) using a particular window length (five, 10, or 20 years, shown in columns). The color of the line indicates which type of weights are used.

As an alternative to the above, the same procedures were carried out except that instead of having non-overlapping time periods and generating one estimate per period, an estimate was generated for each year incorporating all data from a window around that year. This ‘moving window’ method used each observed child-month multiple times and allowed for pooling of information across time while still producing annual estimates. For each year *T*, all child-months were weighted before finding the monthly survival probability for each age group as described in the previous section. Two different kinds of weights were used. In one version, all data within the window were treated equally: for a window of length *x* years, all child-months that occurred between *x*/2 years before time *T* and *x*/2 years after time *T* were assigned a weight of 1, and all other child-months a weight of 0. We refer to these as ‘flat’ weights. In the second version, the weights decreased linearly with time as child-months became further away from *T*, reaching 0 at *x*/2 years on either side of *T*. We refer to these as ‘triangle’ weights. Different length windows can be used, with wider windows providing more pooling of information across time. For this analysis, we tested window lengths of five and 10 years for both variants and 20 years for the triangle-weighted variant. Figure 1 shows the weights that would be applied for estimates in 2000 (top row) and 2005 (bottom row) using a five-year, 10-year, or 20-year window (first, second, and third column, respectively).

### Validation methods

We validate these birth history analysis methods using the following procedure: 

1. For each survey, we calculated ‘true’ under-5 mortality by applying the standard method described above with two-year periods and then linearly interpolating to produce a continuous time-series.

2. Five hundred samples each of sizes 10, 50, 100, 500, and 1,000 women were drawn without replacement from each survey, for a total of 2,500 samples from each survey.

3. Estimates of under-5 mortality were derived for each survey in each of the resulting 2,500 samples using the summary birth history method and each of the complete birth history methods described above.

4. The estimates ( $\hat{{\;}_{5}q_{0}}$) for each of the 2,500 samples from each method were matched to the true under-5 mortality (_5_*q*_0_) by survey and year and then the error, relative error, absolute error, and absolute relative error were calculated as shown in Table 2 for each sample, method, survey, and year. The mean of each error metric was calculated for every sample size and method across all samples and surveys.

**Table 2:** Error metrics

**Metric**	**Formula**	**Interpretation**
Error	$\hat{{\;}_{5}q_{0}}-{\;}_{5}q_{0}$	Measure of bias, in absolute terms.
Relative error	$\frac{\hat{{\;}_{5}q_{0}}-{\;}_{5}q_{0}}{{\;}_{5}q_{0}}$	Measure of bias, in relative terms.
Absolute error	$\left|\hat{{\;}_{5}q_{0}}-{\;}_{5}q_{0}\right|$	Measure of the magnitude of the difference between the estimates and true mortality, in absolute terms.
Absolute relative error	$\left|\frac{\hat{{\;}_{5}q_{0}}-{\;}_{5}q_{0}}{{\;}_{5}q_{0}}\right|$	Measure of the magnitude of the difference between the estimates and true mortality, in relative terms.

**Figure 2:**
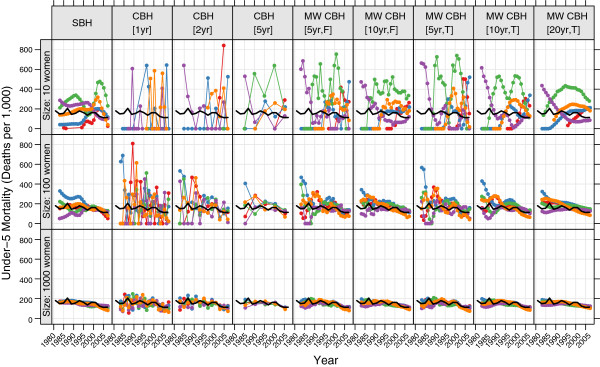
**Example birth history estimates derived from samples of the Zambia 2007 survey.** Each panel shows the ‘true’ under-5 mortality in Zambia (black line) and the under-5 mortality estimates from five samples from this survey. Each column corresponds to a different birth history method, while the rows correspond to the size of the sample (10, 100, and 1000 women). SBH = summary birth history; CBH = complete birth history; MW = moving window; F = flat weights; T = triangle weights; number in brackets gives period or window length.

To illustrate this procedure further, Figure 2 shows examples of the birth history estimates generated from subsamples of one survey (Zambia, 2007). For each method and at three sample sizes (10, 100, and 1000) the birth history series derived from five of the samples are shown alongside the ‘true’ mortality level (shown in black) as calculated from the full sample. Each of the error metrics is based on the comparison of the sample curves (in color) to the ‘true’ mortality curve (in black).

The mean error and mean relative error were intended to indicate whether or not estimates from a given method are biased: since over and underestimates cancel in these metrics, if methods are unbiased (that is, if overestimates and underestimates of the same magnitude are equally likely) the mean error and the mean relative error should be approximately zero. The mean absolute error and mean absolute relative error were intended to capture the extent to which estimates of under-5 mortality can differ from true under-5 mortality; these metrics measure the magnitude of the error, regardless of the direction.

In addition to this overall analysis, we also carried out two stratified analyses. First, country-years were stratified by level of true mortality (<50, 50–100, 100–150, 150–200, >200 deaths per 1,000 births) and the mean of each of the above error metrics was calculated for each method and sample size for each set of country-years. Second, country-years were stratified by the time prior to the survey, 0–1, 2–3, 4–5,..., and 24–25 years prior to the survey, and the mean of each of the above error metrics was calculated for each method and sample size for each set of country-years. These stratified analyses were meant to test if the methods perform consistently well at different levels of mortality and for different lengths of time prior to a survey.

Finally, in order to test how the performance of the complete birth history methods changes when multiple surveys are available and can be pooled, we repeated the above validation procedure on all countries with multiple surveys but pooled both across the survey data when calculating ‘true’ under-5 mortality in step 1 and when estimating birth histories from the 2,500 samples of each survey in step 3. The 2,500 samples were still drawn at the survey level, so for a country with multiple surveys the final number of women is proportional to the number of surveys (e.g., when the sample size for each survey is 10, the total number of women for a given country will be 20 if there are two surveys available, 30 if there are three surveys available, and so on). Consequently, when calculating the mean of each error metric, we stratify by the number of surveys.

All analyses were carried out R, version 2.15.2 [9]. Code is available from the authors upon request.

## Results

### Overall performance

**Figure 3:**
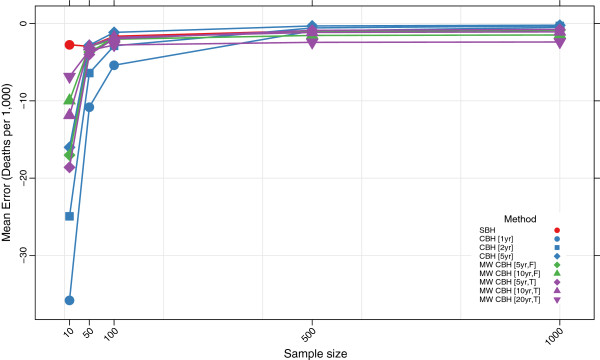
**Mean error for all methods.** Color of marker indicates the birth history method used; shape distinguishes different period or window lengths; SBH = summary birth history; CBH = complete birth history; MW = moving window; F = flat weights; T = triangle weights; number in brackets gives period or window length.

**Figure 4:**
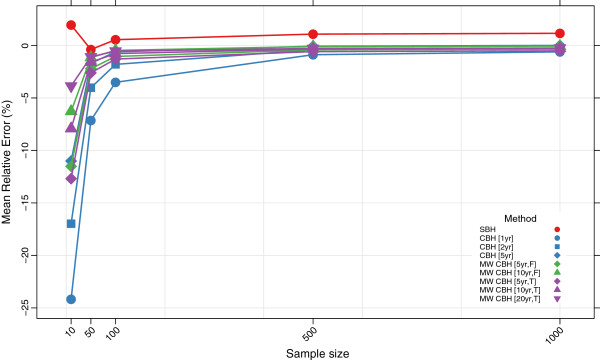
**Mean relative error for all methods.** Color of marker indicates the birth history method used; shape distinguishes different period or window lengths; SBH = summary birth history; CBH = complete birth history; MW = moving window; F = flat weights; T = triangle weights; number in brackets gives period or window length.

**Figure 5:**
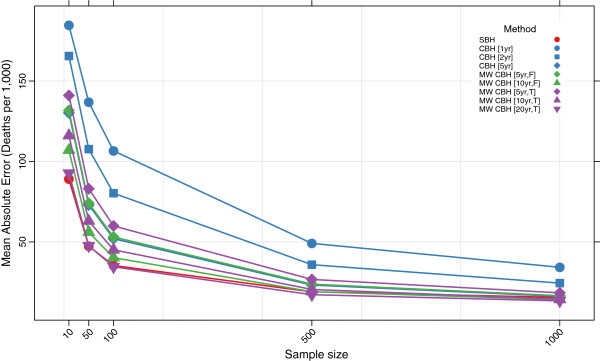
**Mean absolute error for all methods.** Color of marker indicates the birth history method used; shape distinguishes different period or window lengths; SBH = summary birth history; CBH = complete birth history; MW = moving window; F = flat weights; T = triangle weights; number in brackets gives period or window length.

**Figure 6:**
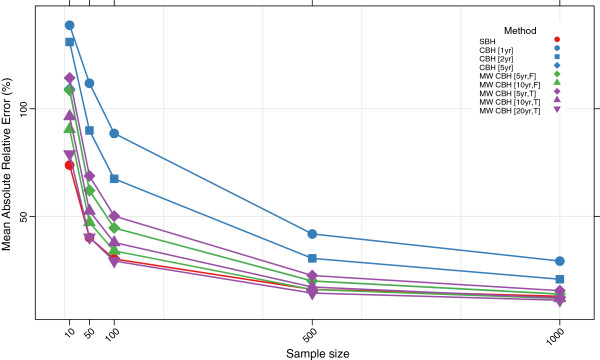
**Mean absolute relative error for all methods.** Color of marker indicates the birth history method used; shape distinguishes different period or window lengths; SBH = summary birth history; CBH = complete birth history; MW = moving window; F = flat weights; T = triangle weights; number in brackets gives period or window length.

Figures 3, 4, 5, and 6 show the mean error, mean relative error, mean absolute error, and mean absolute relative error, respectively, observed for each method at each sample size. Additional file 1: Table S1 also gives these values along with the corresponding 2.5th and 97.5th percentiles.

Overall, all methods are close to unbiased at sample sizes of at least 500, as measured by the mean error and mean relative error. At smaller sample sizes, however, the mean error and mean relative error for the standard complete birth history method becomes noticeably negative, suggesting that these methods tend to underestimate true mortality when sample sizes are small. This tendency is more pronounced when the period length used is smaller: the downward bias observed is more extreme for the one-year estimates than for the five-year estimates, which may reflect the greater pooling of information when longer period lengths are employed. The complete birth history moving window methods follow a similar pattern and are progressively more negatively biased at smaller sample sizes. Similar to the standard methods, for the moving window methods the downward bias is more pronounced when window lengths are shorter. Additionally, for the same window length, there is slightly more downward bias in the triangle weights version than in the flat weights version. In contrast, the summary birth history method appears to be almost unbaised even at small sample sizes.

The mean absolute error and mean absolute relative error of all methods increases noticeably as the sample size decreases. No method performs better on average than 73% error at sample size 10, 40% error at sample size 50, or 29% error at sample size 100. Across all sample sizes there is an ordering of performance among the methods, with moving window complete birth history methods and summary birth history methods generally performing better than standard complete birth history methods. Additionally, within each class of methods, methods with more pooling (e.g., longer periods or windows) have lower error at each sample size than methods with less pooling.

### Stratified by true mortality

**Figure 7:**
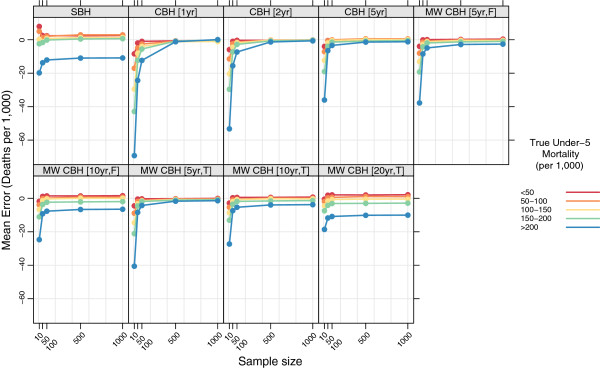
**Mean error for all methods, stratified by true mortality level.** Color indicates level of true mortality; each panel shows results for one particular method; SBH = summary birth history; CBH = complete birth history; MW = moving window; F = flat weights; T = triangle weights; number in brackets gives period or window length.

**Figure 8:**
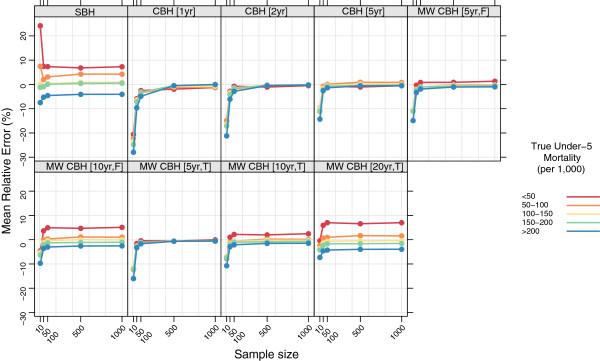
**Mean relative error for all methods, stratified by true mortality level.** Color indicates level of true mortality; each panel shows results for one particular method; SBH = summary birth history; CBH = complete birth history; MW = moving window; F = flat weights; T = triangle weights; number in brackets gives period or window length.

**Figure 9:**
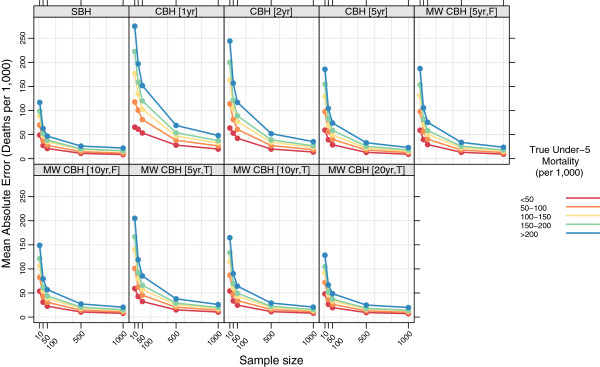
**Mean absolute error for all methods, stratified by true mortality level.** Color indicates level of true mortality; each panel shows results for one particular method; SBH = summary birth history; CBH = complete birth history; MW = moving window; F = flat weights; T = triangle weights; number in brackets gives period or window length.

**Figure 10:**
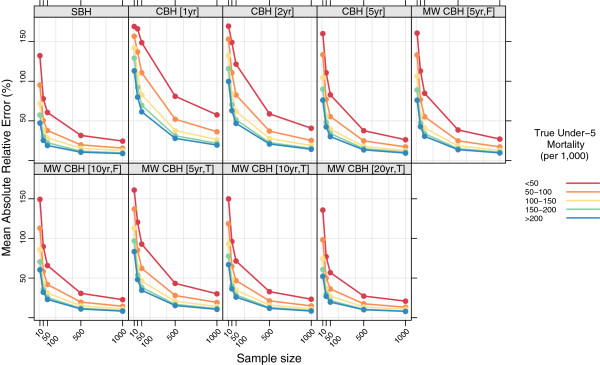
**Mean absolute relative error for all methods, stratified by true mortality level.** Color indicates level of true mortality; each panel shows results for one particular method; SBH = summary birth history; CBH = complete birth history; MW = moving window; F = flat weights; T = triangle weights; number in brackets gives period or window length.

Figures 7, 8, 9, and 10 show the mean error, mean relative error, mean absolute error, and mean absolute relative error, respectively, observed for each method at each sample size stratified by true mortality level. Additional file 1: Table S2 also gives these values along with the corresponding 2.5th and 97.5th percentiles.

For all methods there are some differences in the mean error and mean relative error at different levels of mortality. In general, there is a tendency to underestimate in high mortality settings and to overestimate in low-mortality settings. These differences are most pronounced for the summary birth history method and for the complete birth history methods with long (10- or 20-year) windows. For these methods, the differential is present at all sample sizes and is only slightly attenuated at higher sample sizes compared to the smallest sample sizes. For complete birth history methods with less smoothing, this pattern is less pronounced and is only present at sample sizes smaller than 500.

The magnitude of the error, as measured by the mean absolute error and mean absolute relative error, also varies by level of mortality for all methods. In relative terms (see Figure 10), performance is always poorer when true mortality is lower. This is true for all methods, but the differential is greater in some–notably the standard complete birth history method–than in others and, broadly speaking, increases in magnitude as the sample size decreases. In non-relative terms (see Figure 9), the magnitude of the error is greatest when true mortality is higher. As with the relative measure, the differential in performance between low- and high-mortality situations is greatest for the standard complete birth history method and the moving window birth history method with shorter windows. For all methods, this differential increases as the sample size decreases.

### Stratified by time prior to survey

**Figure 11:**
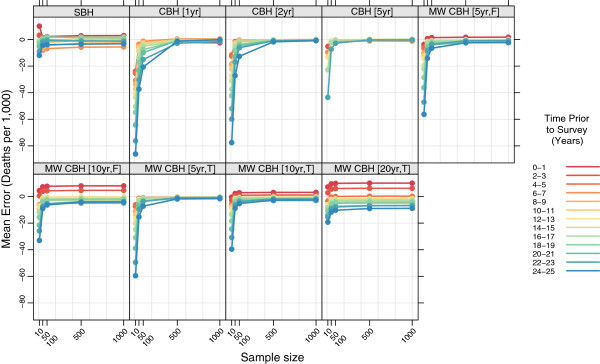
**Mean error for all methods, stratified by time prior to survey.** Color indicates time prior to survey; each panel shows results for one particular method; SBH = summary birth history; CBH = complete birth history; MW = moving window; F = flat weights; T = triangle weights; number in brackets gives period or window length.

**Figure 12:**
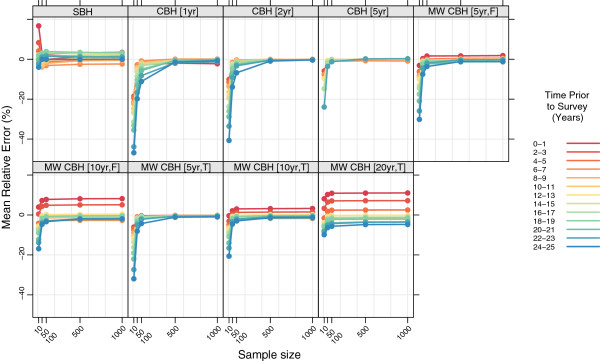
**Mean relative error for all methods, stratified by time prior to survey.** Color indicates time prior to survey; each panel shows results for one particular method; SBH = summary birth history; CBH = complete birth history; MW = moving window; F = flat weights; T = triangle weights; number in brackets gives period or window length.

**Figure 13:**
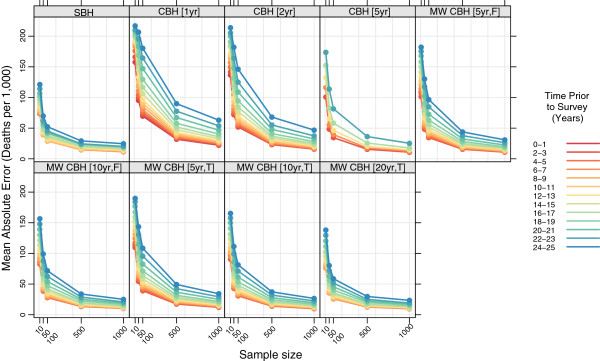
**Mean absolute error for all methods, stratified by time prior to survey.** Color indicates time prior to survey; each panel shows results for one particular method; SBH = summary birth history; CBH = complete birth history; MW = moving window; F = flat weights; T = triangle weights; number in brackets gives period or window length.

**Figure 14:**
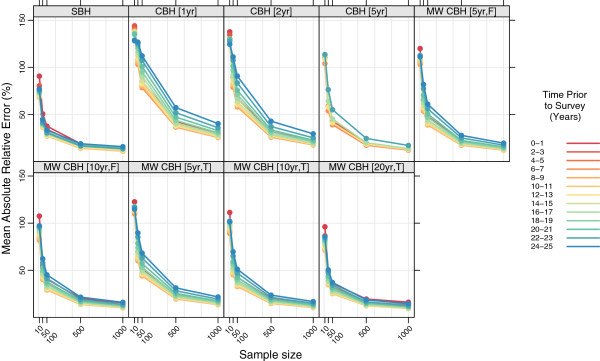
**Mean absolute relative error for all methods, stratified by time prior to survey.** Color indicates time prior to survey; each panel shows results for one particular method; SBH = summary birth history; CBH = complete birth history; MW = moving window; F = flat weights; T = triangle weights; number in brackets gives period or window length.

Figures 11, 12, 13, and 14 show the mean error, mean relative error, mean absolute error, and mean absolute relative error, respectively, observed for each method at each sample size stratified by time prior to survey. Additional file 1: Table S3 also gives these values along with the corresponding 2.5th and 97.5th percentiles.

There are clear differences in the pattern of mean error and mean relative error at different times prior to survey for the summary birth history method, the moving window complete birth history methods with longer windows, and the moving window complete birth history methods with shorter windows, as well as the standard complete birth history methods. There are some differences in mean error and mean relative error between different time periods prior to survey for the summary birth history methods, but while this pattern is consistent across sample sizes, there is not a clear ordering in terms of time periods. In contrast, for complete birth history methods with substantial smoothing (i.e., moving window versions with 10- or 20-year windows), there’s a prominent pattern of over predicting mortality in the most recent period and under predicting mortality in the most distant period. As with the summary birth histories, this pattern is relatively consistent across sample sizes. For the complete birth history methods with less smoothing (i.e., windows and periods of no more than five years) there is little difference in mean error or mean relative error at larger sample sizes, but at smaller sample sizes, the downward bias previously noted in the overall analysis is increasingly concentrated in earlier time periods.

The magnitude of the error, as measured by mean absolute error and mean absolute relative error, varies by time prior to survey for all methods. In absolute terms, all methods perform better for more recent time periods than for more distant time periods. The difference is greatest for the standard complete birth history methods with one- or two-year periods and, in general, decreases as the amount of smoothing increases. The same general pattern is observed in relative terms for most methods, though the difference between the most recent time periods and time periods in the middle of the range are less obvious. In both cases the gap in magnitude of error between different time periods is present at all sample sizes, though it gets somewhat larger as the sample size decreases.

### Multiple surveys

**Figure 15:**
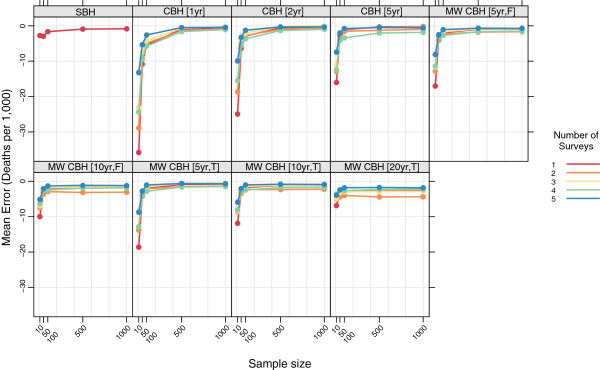
**Mean error for all methods, stratified by the number of pooled surveys.** Color indicates number of surveys; each panel shows results for one particular method; SBH = summary birth history; CBH = complete birth history; MW = moving window; F = flat weights; T = triangle weights; number in brackets gives period or window length.

**Figure 16:**
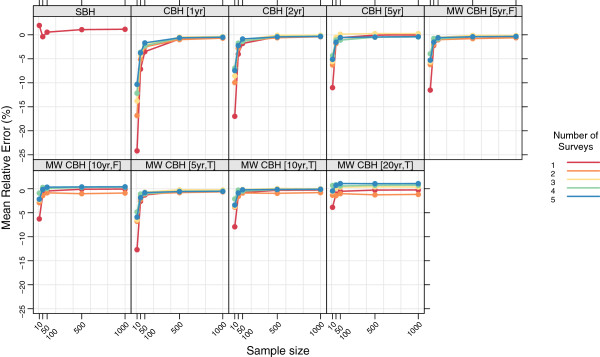
**Mean relative error for all methods, stratified by the number of pooled surveys.** Color indicates number of surveys; each panel shows results for one particular method; SBH = summary birth history; CBH = complete birth history; MW = moving window; F = flat weights; T = triangle weights; number in brackets gives period or window length.

**Figure 17:**
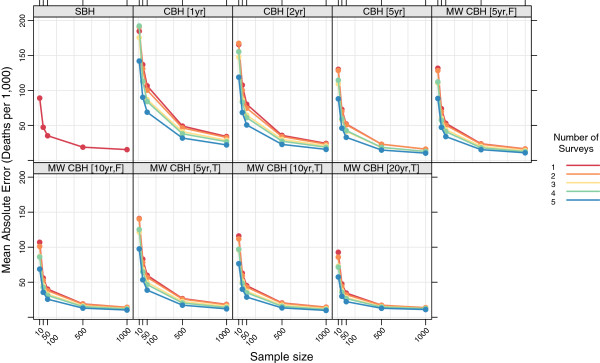
**Mean absolute error for all methods, stratified by the number of pooled surveys.** Color indicates number of surveys; each panel shows results for one particular method; SBH = summary birth history; CBH = complete birth history; MW = moving window; F = flat weights; T = triangle weights; number in brackets gives period or window length.

**Figure 18:**
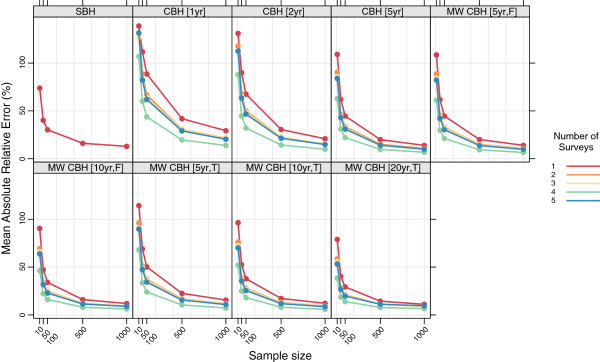
**Mean absolute relative error for all methods, stratified by the number of pooled surveys.** Color indicates number of surveys; each panel shows results for one particular method; SBH = summary birth history; CBH = complete birth history; MW = moving window; F = flat weights; T = triangle weights; number in brackets gives period or window length.

Figures 15, 16, 17, and 18 show the mean error, mean relative error, mean absolute error, and mean absolute relative error, respectively, observed for all methods at each sample size stratified by the number of surveys included. The results shown for a single survey are the same as those shown in Figures 3, 4, 5, and 6 and are included here for comparison. The results shown for multiple surveys are based on complete birth history methods where data are pooled across these multiple surveys within a given country. Additional file 1: Table S4 also gives these values along with the corresponding 2.5th and 97.5th percentiles.

For very small samples, additional surveys appear to alleviate some of the downward bias, as measured by the mean error and mean relative error, exhibited by all of the complete birth history methods. Additionally, there is an obvious decline in the magnitude of the error, as measured by the mean absolute error and the mean absolute relative error, as the number of surveys increases: on average, the mean absolute relative error decreases by 22 percentage points at sample size 10, 20 percentage points at sample size 50, and 15 percentage points at sample size 100 when five surveys are available as compared to a single survey. Both of these effects almost certainly reflect that the overall sample size increases as the number of surveys increases. It is not surprising that the effect of adding additional surveys is in some ways similar to the effect of increasing the sample size in a single survey.

## Discussion

This analysis suggests that all methods of analyzing birth history data perform poorly at sample sizes of fewer than 100 women, with large expected errors and, for some methods, noticeable downward bias. There are large differences in performance between models, however, and even at higher sample sizes (500 and 1000 women), the magnitude of the expected error for many methods is still unacceptably high.

Unfortunately, there is not an obvious ‘best’ method. Overall, summary birth histories and moving window complete birth history methods with very long windows provide estimates with the smallest magnitude error and least bias, especially at the smallest sample sizes. In the case of the former, the better performance may be a result of the models that underlie the method which could, to some extent, constrain more outlying estimates from being generated. In the case of the latter, the better performance, particularly in terms of the expected magnitude of the error, is likely a result of the increased pooling of information across time. These same methods, however, do not perform uniformly across levels of mortality, and in particular, they tend to overestimate in low-mortality settings and underestimate in high-mortality settings. It is likely that the same strengths that underlie the better performance of these models overall are also at least partly responsible for these pitfalls. In the case of the summary birth histories, the models may be constraining final estimates too closely to the mean, biasing unusually low or unusually high estimates toward this mean. In the case of the moving window complete birth history methods, the increased pooling also runs the risk of smoothing out real trends in mortality and biasing the final estimates. Similarly, the moving window complete birth history methods with very long windows do not perform uniformly across time periods prior to the survey: they tend to overestimate in more recent periods and underestimate in more distant periods, and the magnitude of the error increases noticeably the earlier the estimate. Under-5 mortality has generally decreased with time, so it is likely that differences in the level of mortality at different time periods are at least partially driving the differences in performance observed in this analysis at different time periods (the reverse is also possible). Beyond this effect, however, it is also likely that the magnitude of the error is larger in earlier time periods because only the oldest women captured in the survey report children that far in the past and consequently the total number of children observed is smaller in earlier time periods compared to later time periods. The methods with less smoothing (i.e., the complete birth history methods with period or window lengths of no more than five years) are far less problematic with respect to differential bias by level of mortality or time prior to survey, but the magnitude of overall error from these methods is much larger than the other methods.

The results of this analysis suggest that the birth history methods considered are of limited utility for estimating mortality in small samples and, in particular, for making meaningful comparisons among geographic units or strata. Given the value of these types of estimates, however, investment in other data sources may be warranted. In particular, sample registration schemes may be a useful alternative to both surveys, with the problems enumerated here, and full vital registration systems, which are expensive and technically challenging to maintain. Alternatively, research into adapting existing small area methods frequently used in epidemiology and other fields [10,11] for use with birth histories could prove useful. These models explicitly account for unusually high sampling error in estimates derived from small samples and attempt to overcome this challenge by exploiting spatial and temporal relatedness. Several authors have already used birth history data to inform these models, though the focus of these analyses has generally been on the relationship between other factors and mortality and not on prediction of mortality levels for specific areas or subgroups [12-16].

This analysis has several limitations. The stratified analyses by mortality level and time prior to survey do not control for each other, making it difficult to conclusively disentangle the two effects. Further, birth histories, like all survey data, are subject to a number of data errors, including, among others, recall bias and age misreporting. We treat the reported population in each survey as truth and don’t consider the additional effect on error or bias that any of these errors could introduce. It is well documented that these types of errors can impact the reliability of mortality estimates, but future research could consider specifically how these errors interact with the problems due to sample size explicitly considered here. Microsimulation–where synthetic populations are created by simulating births and deaths given set mortality and fertility schedules–could provide useful mechanisms for more fully exploring the issues described here.

Nonetheless, this study boasts several strengths. The use of empirical data, rather than simulated populations, ensures that the mortality and fertility relationships are realistic and representative of the types of scenarios where birth history data are most likely to be collected. Additionally, in contrast to previous research [17,18] which has examined errors in birth history estimates and compared different methods of analyzing birth history data, we estimate error by comparing to a true gold standard (in this case the full sample) rather than using statistical techniques such as the Jackknife to estimate error. Finally, this study compares a large number of different methods for analyzing available data and makes explicit the comparison between these methods at different sample sizes, which should prove useful to analysts deciding between different methods given a particular dataset.

## Conclusions

Overall, the results of this analysis suggest that birth histories in all but the largest of surveys are of limited utility for making subnational estimates or estimates across many strata. Censuses may be more useful for this purpose, having much larger sample sizes, but generally only include summary birth history information if they include birth history information at all. Given the value of subnational and stratified analyses of under-5 mortality and the limitations of the methods examined here, further research into methods for using existing data sources and investment in alternative data sources is warranted. In particular, small area methods, which address the issue of small sample sizes by borrowing strength across geographic units, may be useful when analyzing birth history data at a subnational level.

## Competing interests

The authors declare that they have no competing interests.

## Authors’ contributions

LD-L conceived of the study, carried out the analysis, and wrote the first draft of the manuscript. EG, AF, and HW participated in design of the study, participated in interpretation of results, and edited the manuscript. All authors read and approved the final manuscript.

## Supplementary Material

Additional file 1 **Mean, 2.5th percentile, and 97.5th percentile of the error, relative error, absolute error, and absolute relative error for all methods and sample sizes.** Table S1 gives results for the overall analysis; Table S2 gives results for the analysis stratified by mortality level; Table S3 gives results for the analysis stratified by time prior to survey; and Table S4 gives results for the analysis stratified by number of surveys. Click here for file
